
JOURNAL INFORMATION
==============================
NLM Title Abbreviation: ScientificWorldJournal
Journal ID: ScientificWorldJournal
NLM Journal ID: 101131163
Journal ID: TSWJ

The Scientific World Journal

ISSN: 2356-6140
EISSN: 1537-744X
Publisher: Wiley

ARTICLE INFORMATION
==============================
PMCID: PMC5936535
PMID: 16075148
DOI: 10.1100/tsw.2005.75
Article ID: 824060
Article version: 1
Subjects: Directions in Science

Androgens: New Effects on Proteoglycan Biosynthesis and Its Consequences for Atherosclerosis

Sudhir Krishnankutty 1
Nigro Julie 2
Osman Narin 2 3
Little Peter 2 3 *
1 Stanford University Medical Center, CA 94304-5637, Rm H3554, 300 Pasteur Drive, USA
2 Cell Biology of Diabetes Laboratory, Baker Heart Research Institute, 75 Commercial Rd, Melbourne, Victoria, 3004, Australia
3 Monash University, Faculty of Medicine, Nursing and Health Sciences, Alfred Campus, Prahran, Victoria, Australia
*Peter Little: peter.little@baker.edu.au
Publication date: 2005
Electronic publication date: 2005 Jul 13
Volume: 5
First page: 521
Last page: 526
Received 2005 May 18; Revised 2005 Jul 5; Accepted 2005 Jul 5
Copyright: Copyright © 2005 Krishnankutty Sudhir et al.
Copyright year: 2005
License: This is an open access article distributed under the Creative Commons Attribution License, which permits unrestricted use, distribution, and reproduction in any medium, provided the original work is properly cited.
License URL: https://creativecommons.org/licenses/by/3.0/

Keywords: androgens, atherosclerosis, lipoproteins, proteoglycans, vascular smooth muscle

Funding: National Health and Medical Research Council Alfred Hospital Foundation Diabetes Australia Eli Lilly Endocrinology Research GlaxoSmithKline Australia

==============================
Directions in Science
*TheScientificWorldJOURNAL* (2005) 5, 521–526
ISSN 1537-744X; DOI 10.1100/tsw.2005.75

### Androgens: New Effects on Proteoglycan Biosynthesis and Its Consequences for Atherosclerosis

Krishnankutty Sudhir[1], Julie Nigro[2], Narin Osman[2,3], and Peter J. Little[2,3,*]

*[1]Stanford University Medical Center, Rm H3554, 300 Pasteur Drive, Stanford, CA 94304-5637; [2]Cell Biology of Diabetes Laboratory, Baker Heart Research Institute, 75 Commercial Rd, Melbourne, Victoria, 3004, Australia; [3]Monash University, Faculty of Medicine, Nursing and Health Sciences, Alfred Campus, Prahran, Victoria, Australia*

**E-mail:** ksudhir@alzus.jnj.com; julie.nigro@baker.edu.au; narin.osman@baker.edu.au; peter.little@baker.edu.au

*Received May 18, 2005; Revised July 5, 2005; Accepted July 5, 2005; Published July 13, 2005*

---

**KEYWORDS**: androgens, atherosclerosis, lipoproteins, proteoglycans, vascular smooth muscle

---

### ANDROGENS AND CARDIOVASCULAR DISEASE

Males have a higher risk of cardiovascular disease (CVD) compared to age-matched females despite a postmenopausal increase in risk of CVD in women[1]. While much attention has focused on the protective effect of estrogens, endogenous androgens may be proatherogenic in males. Serum concentrations of testosterone are ten times higher in males (10–35 n*M*) than in females (0.5–2.3 n*M*)[2]. Compared to a vast literature on the vascular effects of estrogens, the role of androgens in atherosclerosis has attracted relatively little attention. Dihydrotestosterone regulates DNA synthesis in human vascular cells[3], while testosterone enhances apoptosis in human endothelial cells (ECs)[4] and worsens endothelial dysfunction in experimental atherosclerosis[5], suggesting that androgens might be proatherogenic. Androgen exposure is associated with increased human monocyte adhesion to ECs, a proatherogenic effect mediated at least in part by an increased EC-surface expression of VCAM-1[6]. Further, Ng et al. have recently shown that androgens up-regulate atherosclerosis-related genes in macrophages from males, but not females[7]. Anabolic androgens accelerate atherosclerosis in primate models[8], markedly reduce HDL-cholesterol, and induce endothelial dysfunction in male body builders[9]. Overall, these studies suggest that androgens adversely influence vascular function and contribute to gender-associated differences in atherogenesis.

Conversely, testosterone is a coronary vasodilator[10] and a study of postmenopausal women showed that endogenous androgens were inversely related to carotid intimal-medial thickness suggesting potential beneficial vascular effects of androgens[11]. Testosterone supplementation in orchidectomized animals with experimental atheroma reduced lesion formation, an effect not observed when the animals were treated simultaneously with the aromatase inhibitor, suggesting that testosterone attenuates early atherogenesis via conversion to estrogens by the enzyme aromatase expressed in the vessel wall[12]. We have shown that DHEA inhibits human vascular smooth muscle cell (vSMC) proliferation by a

***Corresponding author:** Associate Professor Peter J. Little, Head, Cell Biology of Diabetes Laboratory, Baker Heart Research Institute, St Kilda Road Central, PO Box 6492, Melbourne, Vic 8008, AUSTRALIA; Tel: + 61 3 8532 1203/Fax: + 61 3 8532 1100
©2005 with author.

mechanism independent of either androgen receptors (ARs) or estrogen receptors (ERs), presumably via a DHEA-specific receptor that involves ERK1 signaling pathways[13]. Further, we have also shown that DHEA increases EC proliferation *in vitro* by mechanism(s) independently of either AR or ER and *in vivo* enhances large- and small-vessel endothelium-dependent vasodilation in postmenopausal women[14]. Thus, the role of androgens on atherogenesis is potentially complex, with both deleterious and protective effects, and requires further study. One of the hypotheses of atherogenesis known as “Response to Retention”[15], which is based on the binding and retention of atherogenic lipoproteins, has recently risen to prominence[16,17]. This article focuses on the contribution of proteoglycan synthesis and structure to atherogenesis and new findings on the action of androgens on proteoglycan biosynthesis in vSMC.

### MODIFICATION OF PROTEOGLYCANS AND THE “RESPONSE TO RETENTION” HYPOTHESIS

The two processes that can alter the vascular proteoglycan compartment in a manner that may increase the propensity for the initiation of atherosclerosis are an alteration in the level of expression and the mix of proteoglycans or a change in the biochemical fine structure of the glycosaminoglycan (GAG) chains on the proteoglycans that are associated with apolipoprotein binding (Fig. 1). The biochemical changes in GAGs that can alter binding to apolipoproteins have recently been reviewed in detail[18]. At this time, the major biochemical property of proteoglycans that can be most clearly associated, at least in *in vitro* models, with the binding of apolipoproteins is the length of the GAG chains[18]. The interaction between LDL and proteoglycans can be studied *in vitro* using a Gel Mobility Shift Assay (GMSA) that most usually measures the interaction between metabolically radiolabeled proteoglycans synthesized by vSMCs and a range of concentrations of normal human LDL by electrophoretically separating the bound from free proteoglycan on a flat bed gel arrangement allowing for determination of a curve equivalent to a binding isotherm[19]. This technique allows for the vSMCs to be treated with hormones, growth factors, and drugs, and for an assessment to be made of the effect of such treatments on the characteristics by which the isolated and radiolabeled proteoglycans bind to LDL. The proteoglycans can be assessed for changes in size by gradient SDS-PAGE and more rigorously by size-exclusion chromatography. Changes in the size of the proteoglycans reside only in changes in the size of the GAG chains and not in the size of the proteoglycan core proteins. Apolipoproteins bind only to the GAG chains and not to the core proteins[20]. Changes in size of the proteoglycan can be further assessed by chemically cleaving the chains from the core proteins and assessing the size of the free chains. A further technique, based on the assumption that the chain synthesizing mechanisms in the cell influences all chondroitin/dermatan sulfate proteoglycans similarly, is to provide the cells with excess xyloside that acts as a “false acceptor” leading to the syntheses of small GAGs without core proteins. We recently demonstrated that treatment of vSMCs with the proatherogenic growth factor, Transforming Growth Factor (TGF)-β, leads to the synthesis of proteoglycans that are larger than the respective controls, that the free chains released from these proteoglycans are also larger, and that the xyloside-initiated GAGs from TGF-β–treated cells were also larger than their respective controls[20]. Most interestingly and convincingly, we demonstrated that LDL binding studies by GMSA revealed that for intact proteoglycans, free chains from proteoglycans, and xyloside-initiated GAGs, the products from the cells treated with TGF-β all showed enhanced binding to LDL[20].

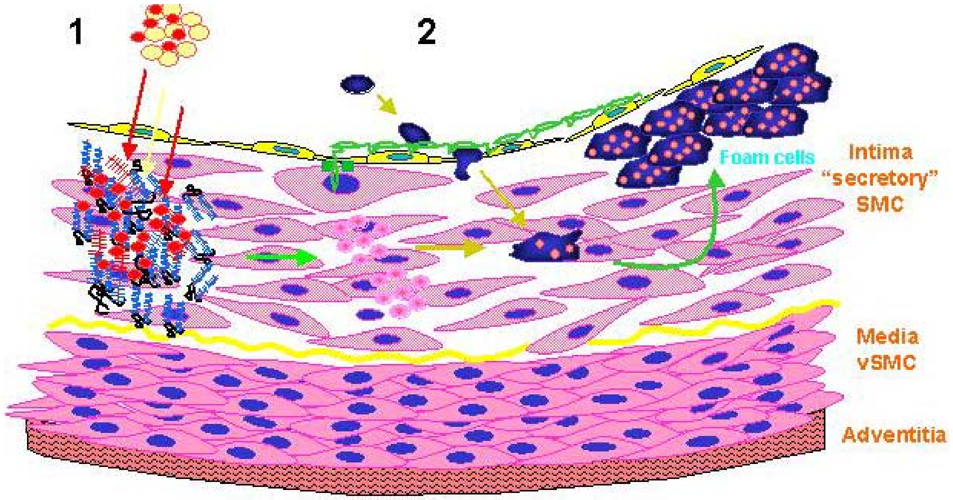

**FIGURE 1**. Stylized presentation of the two processes of the initiation of atherosclerosis. (1) The binding and retention of atherogenic lipoproteins (LDL) to modified proteoglycans; (2) the binding and adhesion of monocytes/macrophages to the activated endothelium. See text for further details.

Platelet-derived growth factor (PDGF)[21], oxidized LDL[22], fatty acids[23], and angiotensin II[24] increase the size of proteoglycans produced by vSMCs and these show greater binding to LDL. In the current context, we have recently determined the effects of androgens on proteoglycan biosynthesis in vSMCs and the properties of the proteoglycans for binding to human LDL.

### ANDROGENS AND PROTEOGLYCAN BIOSYNTHESIS IN VASCULAR SMOOTH MUSCLE

Peroxisome Proliferator Activator Receptors (PPARs) are members of the steroid superfamily of receptors that are transcription factors and are known to have multiple effects on proteoglycan synthesis in vSMCs[25,26]. Androgens are also members of this family and with the controversial situation with respect to hormones and CVD, we evaluated the effect of androgens in our model of proteoglycan synthesis and LDL binding. We investigated the effect of specific androgens, androstenedione, dihydrotestosterone, and testosterone on proteoglycan biosynthesis in human vSMCs derived from internal mammary arteries[27]. In these experiments, human vSMCs were metabolically labeled with [$^{35}$S]-sulfate or [$^{35}$S]-methionine/cysteine to assess GAGs or proteoglycan core protein synthesis, respectively. The electrophoretic migration of radiolabled proteoglycans (as a surrogate measure of molecular size and, thus, GAG chain length) was assessed by SDS-PAGE and proteoglycan-LDL interactions were assessed using LDL affinity columns. Treatment of vSMCs with androstenedione (100 n*M*), dihydrotestosterone (10 n*M*), or testosterone (100 n*M*) increased [$^{35}$S]-sulfate incorporation into GAGs by 24.8% ($p < 0.05$), 22% ($p < 0.05$), and 32.5% ($p < 0.05$), respectively. Treatment of vSMCs with testosterone did not alter [$^{35}$S]-methionine/cysteine incorporation into proteoglycan core protein synthesis, indicating that the effect of testosterone was associated with an increase in GAG length. Dihydrotestosterone (10 n*M*) and testosterone (100 n*M*) treatment of vSMCs resulted in the synthesis of biglycan and decorin that showed reduced electrophoretic mobility by SDS-PAGE, indicating an increase in GAG length. The effect of testosterone treatment on [$^{35}$S]-sulfate incorporation and GAG length was reversed by the pretreatment of vSMCs with flutamide (1 $\mu$*M*), an androgen receptor antagonist. Based on the ability of androgens to alter the properties of proteoglycans, we assessed the properties of proteoglycans from androgen-treated cells binding to LDL. Proteoglycans from vSMCs treated with testosterone showed modest (11%), but statistically significant ($p < 0.01$), higher binding capacity to LDL compared to proteoglycans from untreated cells[27].

### MECHANISM OF ACTION OF ANDROGENS ON PROTEOGLYCAN SYNTHESIS

GAG size is controlled by a family of enzymes that act in the Golgi to grow the developing chain on the proteoglycan core protein[28]. As noted above, the effect of the cell treatments can also be demonstrated on the synthesis of the xyloside-initiated GAGs, indicating that the process is independent of core proteins[20]. It may be that androgens can induce the expression of the enzymes responsible for GAG elongation in vSMC. The effects of androgens are quite subtle[27]. In the context of the long development of atherosclerosis, it would be argued that small effect over a long period might have an impact in such a chronic disease process. However, such effects presently remain, most likely, too subtle to follow experimentally with demonstrations of mechanism. We have recently identified a number of tools that have profound effects on GAG synthesis in vSMC and we are hopeful that these tools may provide us with the entrée to this area of investigation and allow for the identification and study of the rate limiting enzyme(s) and their control by pharmacological and therapeutic agents[18]. In a related context, we showed that the inhibition of GAG synthesis by the fibrate fenofibrate was accompanied by an action of the drug to stimulate the synthesis of proteoglycan core proteins[29]. Thus, steroid family receptors have multiple effects on vascular proteoglycans and unravelling the roles of these agents may not only divulge interesting effects on metabolism relevant to CVD, but also on direct vascular actions.

### CONCLUSIONS

Our evaluation of the effects of androgens on proteoglycan biosynthesis and the interaction with LDL showed that androgen treatment of human vSMCs increased proteoglycan synthesis and GAG length and this resulted in an increase in binding to LDL. The effect of testosterone on vascular proteoglycans was dependent on the testosterone receptor because the response was inhibited by the receptor antagonist flutamide. There are several lines of evidence that show that testosterone or dihydrotestosterone stimulates proteoglycan biosynthesis in the prostate[30], testis[31], cartilage[32], and submandibular glands[33], however, the mechanism(s) by which atherogenic effects of androgens are mediated in vSMCs are unclear. One possible explanation for the atherogenic effects of androgens involves direct effects on atherogenesis and progression at the level of the arterial intima. Risk factors for CVD interact negatively in a synergistic manner, thus, a small change in proteoglycans induced by elevated androgens in addition to the presence of one or more coronary risk factors, may render males more susceptible to atherosclerosis than females.

### ACKNOWLEDGMENTS

Recent work in the laboratory has been supported by the National Health & Medical Research Council of Australia, Alfred Hospital Foundation, Diabetes Australia Research Trust grants, Eli Lilly Endocrinology Research Awards (to PJL), and project funding from GlaxoSmithKline Australia.

### REFERENCES

1. Gorodeski, G. and Utian, W. (1994) Epidemiology and risk factors of cardiovascular disease in postmenopausal women. In *Treatment of the Postmenopausal Woman: Basic and Clinical Aspects.* Lobo, R., Ed. Raven Press, New York. pp. 199–221.
2. Vermeulen, A., Verdonck, L., and Kaufman, J.M. (1999) A critical evaluation of simple methods for the estimation of free testosterone in serum. *J. Clin. Endocrinol. Metab.* **84,** 3666–3672.

3. Somjen, D., Kohen, F., Jaffe, A., Amir-Zaltsman, Y., Knoll, E., and Stern, N. (1998) Effects of gonadal steroids and their antagonists on DNA synthesis in human vascular cells. *Hypertension* **32,** 39–45.
4. Ling, S., Dai, A., Williams, M.R., Myles, K., Dilley, R.J., Komesaroff, P.A., and Sudhir, K. (2002) Testosterone (T) enhances apoptosis-related damage in human vascular endothelial cells. *Endocrinology* **143,** 1119–1125.
5. Hutchison, S.J., Sudhir, K., Chou, T.M., Sievers, R.E., Zhu, B.Q., Sun, Y.P., Deedwania, P.C., Glantz, S.A., Parmley, W.W., and Chatterjee, K. (1997) Testosterone worsens endothelial dysfunction associated with hypercholesterolemia and environmental tobacco smoke exposure in male rabbit aorta. *J. Am. Coll. Cardiol.* **29,** 800–807.
6. McCrohon, J.A., Jessup, W., Handelsman, D.J., and Celermajer, D.S. (1999) Androgen exposure increases human monocyte adhesion to vascular endothelium and endothelial cell expression of vascular cell adhesion molecule-1. *Circulation* **99,** 2317–2322.
7. Ng, M.K., Quinn, C.M., McCrohon, J.A., Nakhla, S., Jessup, W., Handelsman, D.J., Celermajer, D.S., and Death, A.K. (2003) Androgens up-regulate atherosclerosis-related genes in macrophages from males but not females: molecular insights into gender differences in atherosclerosis. *J. Am. Coll. Cardiol.* **42,** 1306–1313.
8. Obasanjo, I.O., Clarkson, T.B., and Weaver, D.S. (1996) Effects of the anabolic steroid nandrolone decanoate on plasma lipids and coronary arteries of female cynomolgus macaques. *Metabolism* **45,** 463–468.
9. Ebenbichler, C.F., Sturm, W., Ganzer, H., Bodner, J., Mangweth, B., Ritsch, A., Sandhofer, A., Lechleitner, M., Foger, B., and Patsch, J.R. (2001) Flow-mediated, endothelium-dependent vasodilatation is impaired in male body builders taking anabolic-androgenic steroids. *Atherosclerosis* **158,** 483–490.
10. Chou, T.M., Sudhir, K., Hutchison, S.J., Ko, E., Amidon, T.M., Collins, P., and Chatterjee, K. (1996) Testosterone induces dilation of canine coronary conductance and resistance arteries in vivo. *Circulation* **94,** 2614–2619.
11. Bernini, G.P., Moretti, A., Sgro, M., Argenio, G.F., Barlascini, C.O., Cristofani, R., and Salvetti, A. (2001) Influence of endogenous androgens on carotid wall in postmenopausal women. *Menopause* **8,** 43–50.
12. Nathan, L., Shi, W., Dinh, H., Mukherjee, T.K., Wang, X., Lusis, A.J., and Chaudhuri, G. (2001) Testosterone inhibits early atherogenesis by conversion to estradiol: critical role of aromatase. *Proc. Natl. Acad. Sci. U. S. A.* **98,** 3589–3593.
13. Williams, M.R., Ling, S., Dawood, T., Hashimura, K., Dai, A., Li, H., Liu, J.P., Funder, J.W., Sudhir, K., and Komesaroff, P.A. (2002) Dehydroepiandrosterone inhibits human vascular smooth muscle cell proliferation independent of ARs and ERs. *J. Clin. Endocrinol. Metab.* **87,** 176–181.
14. Calkin. A.C., Sudhir, K., Honisett, S., Williams, M.R., Dawood, T., and Komesaroff, P.A. (2002) Rapid potentiation of endothelium-dependent vasodilation by estradiol in postmenopausal women is mediated via cyclooxygenase 2. *J. Clin. Endocrinol. Metab.* **87,** 5072–5075.
15. Williams, K.J. and Tabas, I. (1995) The response-to-retention hypothesis of early atherogenesis. *Arterioscler. Thromb. Vasc. Biol.* **15,** 551–561.
16. Skalen, K., Gustafsson, M., Rydberg, E.K., Hulten, L.M., Wiklund, O., Innerarity, T.L., and Boren, J. (2002) Subendothelial retention of atherogenic lipoproteins in early atherosclerosis. *Nature* **417,** 750–754.
17. Staels, B. (2002) Cardiovascular biology: a cholesterol tether. *Nature* **417,** 699–701.
18. Ballinger, M., Nigro, J., Frontanilla, K., Dart, A., and Little, P. (2004) Regulation of glycosaminoglycan structure and atherogenesis. *Cell. Mol. Life Sci.* **61,** 1296–1306.
19. Camejo, G. (1982) The interaction of lipids and lipoproteins with the intercellular matrix of arterial tissue: its possible role in atherogenesis. *Adv. Lipid Res.* **19,** 1–53.
20. Little, P.J., Tannock, L., Olin, K.L., Chait, A., and Wight, T.N. (2002) Proteoglycans synthesized by arterial smooth muscle cells in the presence of transforming growth factor-beta1 exhibit increased binding to LDLs. *Arterioscler. Thromb. Vasc. Biol.* **22,** 55–60.
21. Schonherr, E., Jarvelainen, H.T., Sandell, L.J., and Wight, T.N. (1991) Effects of platelet-derived growth factor and transforming growth factor-beta 1 on the synthesis of a large versican-like chondroitin sulfate proteoglycan by arterial smooth muscle cells. *J. Biol. Chem.* **266,** 17640–17647.
22. Chang, M.Y., Potter-Perigo, S., Tsoi, C., Chait, A., and Wight, T.N. (2000) Oxidized low density lipoproteins regulate synthesis of monkey aortic smooth muscle cell proteoglycans that have enhanced native low density lipoprotein binding properties. *J. Biol. Chem.* **275,** 4766–4773.
23. Olsson, U., Bondjers, G., and Camejo, G. (1999) Fatty acids modulate the composition of extracellular matrix in cultured human arterial smooth muscle cells by altering the expression of genes for proteoglycan core proteins. *Diabetes* **48,** 616–622.
24. Figueroa, J.E. and Vijayagopal, P. (2002) Angiotensin II stimulates synthesis of vascular smooth muscle cell proteoglycans with enhanced low density lipoprotein binding properties. *Atherosclerosis* **162,** 261–268.
25. Nigro, J., Dilley, R.J., and Little, P.J. (2002) Differential effects of gemfibrozil on migration, proliferation and proteoglycan production in human vascular smooth muscle cells. *Atherosclerosis* **162,** 119–129.
26. Tannock, L.R., Little, P.J., Tsoi, C., Barrett, P.H.R., Wight, T.N., and Chait, A. (2004) Thiazolidinediones reduce the LDL binding affinity of non-human primate vascular cell proteoglycans. *Diabetologia* **47,** 837–843.
27. Hashimura, K., Sudhir, K., Nigro, J., Ling, S., Williams, M.R., Komesaroff, P.A., and Little, P.J. (2005) Androgens stimulate human vascular smooth muscle cell proteoglycan biosynthesis and increase lipoprotein binding. *Endocrinology* **146,** 2085–2090.
28. Prydz, K. and Dalen, K.T. (2000) Synthesis and sorting of proteoglycans. *J. Cell Sci.* **113(Pt 2),** 193–205.

29. Nigro, J., Ballinger, M., Dilley, R., Jennings, G., Wight, T., and Little, P. (2004) Fenofibrate modifies human vascular smooth muscle proteoglycans and reduces LDL binding. *Diabetologia* **47,** 2105–2113.
30. Terry, D.E. and Clark, A.F. (1996) Influence of testosterone on chondroitin sulphate proteoglycan in the rat prostate. *Biochem. Cell Biol.* **74,** 645–651.
31. Raychoudhury, S.S., Blackshaw, A.W., and Irving, M.G. (1993) Rat Sertoli cell extracellular matrix regulates glycosaminoglycan synthesis by peritubular myoid cells in vitro. *Mol. Reprod. Dev.* **35,** 151–158.
32. Franchimont, P. and Bassleer, C. (1991) Effects of hormones and local growth factors on articular chondrocyte metabolism. *J. Rheumatol. Suppl.* **27,** 68–70.
33. Kofoed, J.A., Tumilasci, O.R., Curbelo, H.M., Fernandez Lemos, S.M., Arias, N.H., and Houssay, A.B. (1990) Effect of testosterone on proteoglycans in the submandibular glands of the rat. *J. Biol. Buccale* **18,** 255–259.

**This article should be referenced as follows:**

Sudhir, K., Nigro, J., Osman, N., and Little, P.J. (2005) Androgens: new effects on proteoglycan biosynthesis and its consequences for atherosclerosis. *TheScientificWorldJOURNAL* **5**, 521–526.

**Handling Editor:**

M. Gotte, Principal Editor for *Cell Biology* and Editorial Board Member for *Biochemistry* and *Molecular Biology* — domains of *TheScientificWorldJOURNAL*.

### BIOSKETCH

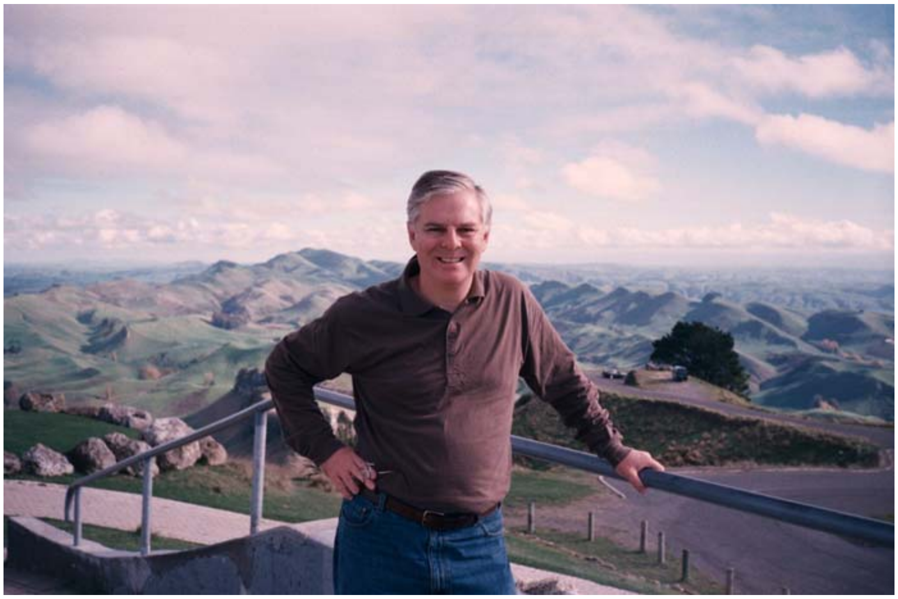

**Peter Little, B.Pharm., M.Sc., Ph.D.,** Associate of the Securities Institute of Australia, is Head of the Cell Biology of Diabetes Laboratory at the Baker Heart Research Institute in Melbourne, Australia; an Associate Professor in the Department of Medicine at Monash University; and Adjunct Professor at RMIT University, Melbourne, Australia. Professor Little is also the Chairman of the Board of Directors and National President of Diabetes Australia, the not-for-profit peak body for diabetes in Australia. Professor Little's laboratory is studying the pathways through which inhibition of the proatherogenic actions of vascular growth factors and hormones might lead to the discovery of an agent capable of preventing atherosclerosis.
